# The VAPB Axis Precisely Coordinates the Timing of Motoneuron Dendritogenesis in Neural Map Development

**DOI:** 10.21203/rs.3.rs-5684747/v1

**Published:** 2024-12-31

**Authors:** Daichi Kamiyama, Yuri Nishida, Rie Kamiyama, Anthony Sego, George Vining, Kathy Bui, Miyuki Fitch, Hy Do, Oshri Avraham, Takahiro Chihara

**Affiliations:** University of Georgia; University of Georgia; University of Georgia; University of Georgia; University of Georgia; University of Georgia; University of Georgia; University of Georgia; University of Georgia; Hiroshima University

## Abstract

In *Drosophila* motoneurons, spatiotemporal dendritic patterns are established in the ventral nerve cord. While many guidance cues have been identified, the mechanisms of temporal regulation remain unknown. Previously, we identified the actin modulator Cdc42 GTPase as a key factor in this process. In this report, we further identify the upstream factors that activate Cdc42. Using single-cell genetics, FRET-based imaging, and biochemical techniques, we demonstrate that the guanine nucleotide exchange factor Vav is anchored to the plasma membrane via the Eph receptor tyrosine kinase, enabling Cdc42 activation. VAMP-associated protein 33 (Vap33), an Eph ligand supplied non-cell-autonomously, may induce Eph autophosphorylation, initiating downstream signaling. Traditionally known as an ER-resident protein, Vap33 is secreted extracellularly at the onset of Cdc42 activation, acting as a temporal cue. In humans, VAPB—the ortholog of Vap33—is similarly secreted in the spinal cord, and its dysregulation leads to amyotrophic lateral sclerosis type 8 (ALS8) and spinal muscular atrophy (SMA). Our findings provide a framework linking VAPB signaling to motor circuitry formation in both health and disease.

## Introduction

Precise synaptic connections are critical for proper brain function during development. The brain’s functionality hinges on unique patterns of connections between neuron populations, which form distinct neural maps ^1–4^. The creation of these maps depends on the correct patterning of axons and dendrites, with axons targeting specific zones or laminae to connect with the dendrites of postsynaptic neurons. This process is observed in multiple brain regions, including the cerebral cortex, retina and superior colliculus, thalamus, and hippocampus ^5–11^. Our understanding of the cellular principles that shape neural maps has greatly advanced through studies of sensorimotor systems ^10–15^. Recent connectomic research in model organisms, such as *Drosophila melanogaster*, has further revealed individual neurons involved in assembling these intricate systems ^16–28^. Additionally, a wide range of genetic tools in *Drosophila* has greatly facilitated the discovery of genes essential for organizing and developing neuronal connections. One of the most advanced areas in this research is the study of the adult olfactory map in *Drosophila*. The assembly of this neural map begins with the dendritic outgrowth and guidance of olfactory projection neurons (PNs), which lay the groundwork for a prototypical map ^29–33^: Before the presynaptic olfactory receptor neuron (ORN) axons innervate the region, PNs establish their dendrites in specific territories, predefining the framework for future connections. This process has sparked significant interest in understanding how positional cues guide PN dendrites. As a result, numerous cell-surface proteins, including key guidance molecules, have been identified as critical in directing the formation of dendritic arbors and synaptic connections ^34–37^. Although the mechanisms by which dendritic arbors navigate toward their final target fields are well understood, the initial processes governing how dendrites extend into these target areas remain largely unknown.

Hints of initial dendritic outgrowth have emerged from studies on motoneurons (MNs) in the embryonic ventral nerve cord (VNC), where approximately 36 MNs reside in each hemi-segment ^38–43^. These MNs extend their dendrites into specific regions of the neuropil, creating a myotopic map, a prototypical neural map ^44–55^. Remarkably, the outgrowth and guidance of dendritic arbors in this system occur independently of presynaptic partners, muscles, and glia ^44^. This process is largely regulated by a set of guidance molecules, including the Slit-Robo and Netrin-Frazzled signaling pathways ^45–47^. In mutants lacking functional Robo or Fra receptors, dendritic arbors are misdirected. However, a closer examination reveals that while higher-order branches are significantly affected, the primary (1°) and secondary (2°) branches remain largely intact, resembling wild-type branches. This observation suggests the existence of an additional program specifically promoting the outgrowth of these initial 1° and 2° branches. As the saying goes, “A journey of a thousand miles begins with a single step,” and understanding this initial step is key to unraveling the formation of this neural map and the locomotion circuit it governs.

During embryonic development, dendrites extend from the axon shaft, initiating with filopodia-like structures that emerge from the proximal region of the shaft. These filopodia eventually stabilize and form the primary (1°) and secondary (2°) branches. Interestingly, in most motoneurons, regardless of their lineage or birth time, these filopodia appear synchronously around 13 hours after egg laying (AEL) ^50–53, 55–57^. This simultaneous extension, rather than a sequential outgrowth, seems to be a key feature of motoneuron development and has also been observed in the leg motoneurons of adult flies ^58^. To investigate the molecular mechanisms underlying filopodia extension, we focused on the most well-characterized motoneuron, the anterior corner cell (aCC) motoneuron. Our previous work has explored the role of Cdc42, a small Rho GTPase, which is known to be a central regulator of filopodia formation ^51^. Cdc42 alternates between two conformations: an “on” state when bound to GTP and an “off” state when bound to GDP. Guanine nucleotide exchange factors (GEFs) and GTPase-activating proteins (GAPs) regulate Cdc42 by driving it into these active or inactive states, respectively ^59, 60^. Numerous imaging studies using Cdc42 biosensors have demonstrated that Cdc42 is activated at the base of filopodia in various cell types during their formation ^61–63^. Similarly, in the aCC motoneuron, we observed Cdc42 activation at the precise locations where dendritic filopodia emerge ^51^. Notably, this activation begins exactly at 13:00 AEL, coinciding with the onset of dendritic filopodia formation. This temporal synchronization suggests that Cdc42 plays a critical role as a timing cue for filopodia emergence. Supporting this hypothesis, *Cdc42* loss-of-function mutants showed a significant reduction in dendritic filopodia in aCC motoneurons, further emphasizing its importance in the process.

A similar loss-of-dendrite phenotype in *Cdc42* mutants was observed in other motoneurons ^51^, suggesting that the regulation of Cdc42 activation is broadly conserved across motoneurons. However, the precise molecular mechanisms controlling Cdc42 activation remain unclear. To address this, we performed a genetic screen using RNA interference (RNAi) to knock down the function of Cdc42 guanine nucleotide exchange factors (GEFs). Through this screening, we identified Vav, a member of the Dbl family of GEFs, as a key activator of Cdc42. Further genetic and biochemical assays revealed that the Eph receptor tyrosine kinase localizes Vav to the plasma membrane, thereby facilitating Cdc42 activation. Additionally, we discovered that VAMP-associated protein 33 (Vap33) functions as a ligand for the Eph receptor in this pathway. Vap33 is a type IV transmembrane protein typically localized to the endoplasmic reticulum (ER) ^64, 65^. However, our imaging analyses indicate that during the time of Cdc42 activation, Vap33 becomes exposed extracellularly in the VNC. This extracellular exposure may enable the Eph-Vav-Cdc42 signaling cascade to initiate filopodia formation in motoneurons in a timely manner. Importantly, dysfunction of VAPB, the human ortholog of Vap33, has been implicated in the development of amyotrophic lateral sclerosis type 8 (ALS8) and spinal muscular atrophy (SMA) ^66–68^. Our findings further suggest a potential role for VAPB signaling in establishing motor circuitry, with disruptions in this pathway possibly contributing to early-onset forms of ALS and SMA.

## Results

### The Rho Family GEF Vav Has a Cell-Autonomous Function in aCC Dendritogenesis

**Figure 1:**
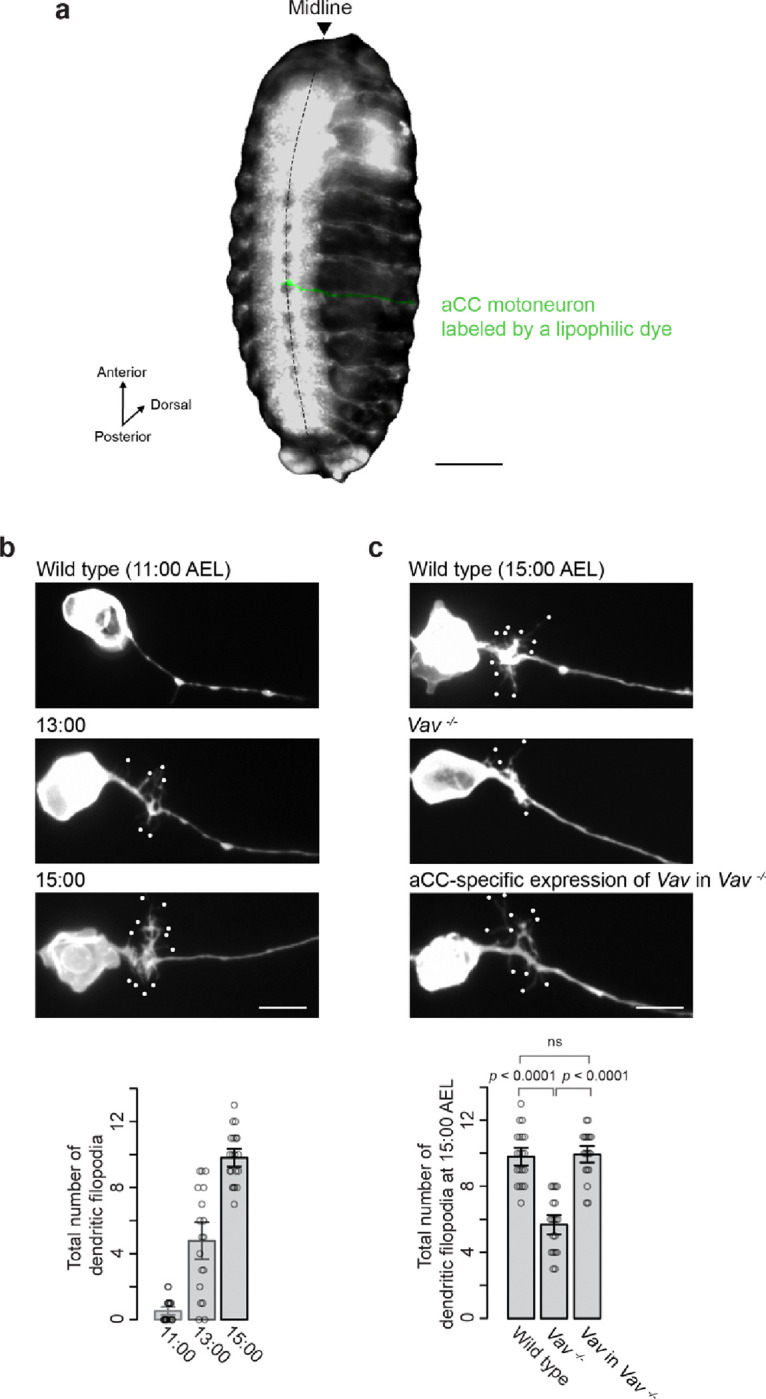
The *Vav*Gene is Essential for Dendritic Filopodia Outgrowth (**a**) A representative image of the aCC motoneuron (green) in abdominal segment 4 (A4) of an embryo at 15:00 after egg laying (AEL), shown in a ventral view with anterior oriented upwards. (**b**) Top: Normal development of the aCC motoneuron in wild-type embryos at various developmental stages, as indicated in each panel. Images are oriented with anterior upwards and dorsal to the right. Dots mark the tips of dendritic filopodia longer than 1 mm, demonstrating the growth of dendritic filopodia over time. **Bottom:** Quantification of dendritic filopodia number at different developmental stages. Data are expressed as mean ± SEM, based on the analysis of 18–20 neurons at each stage. (**c**) **Top:** Representative images of dendritic outgrowth in wild-type, *Vav* knockout (*Vav*^*−/−*^), and aCC-specific expression of *Vav* in *Vav*^*−/−*^ embryos at 15:00 AEL. **Bottom:** Quantification of dendritic filopodia number for each genotype. Statistical significance was determined by one-way ANOVA (*F*_*2,55*_ = 44, *p* = 3.9 × e^−12^, followed by Tukey’s multiple comparison test. “ns” indicates p > 0.05, indicating no significant difference. The analysis included 19–20 neurons per genotype. Analyses of dendritic filopodia in all other figures are also shown at 15:00 AEL. Scale bars: 100 mm in (a); 5 mm in (b) and (c).

aCC motoneurons are located in an abdominal segment of the VNC (Fig. 1a). These aCC motoneurons initiate axon extension at 9 hours after egg laying (AEL) (**Extended Data** Fig. 1a). Our primary focus in this study is the initial step of dendritic outgrowth. To explore this, we conducted a detailed characterization of their dendritic processes (Fig. 1b). To accurately characterize the dendritic processes in these neurons, we employed a retrograde labeling approach ^69^. In this method, a lipophilic dye was injected into the axon tips of fixed samples at different developmental time points. This technique enables high-density membrane labeling, allowing for the clear resolution of each dendritic branch. Observations made before 11:00 AEL revealed a minimal presence of filopodia along the axons. By 13:00 AEL, filopodia began to emerge in the proximal region of the axons. Previous live imaging has shown that these filopodia exhibit dynamic behavior around 13:00 AEL, frequently extending and retracting. By 15:00 AEL, these structures become more stable, allowing for reliable quantification. By 17:00 AEL, the transition from filopodia to dendritic branches is complete, with these filopodia-like structures having matured into dendritic branches forming tertiary or higher-order branches and establishing functional synaptic connections ^49, 50^. We refer to these filopodia-like structures as “dendritic filopodia” and identify the period from 13:00 to 15:00 AEL as the onset of “dendritogenesis.” Our previous research showed that *Cdc42* mutations resulted in a significant reduction in the number of dendritic filopodia at 15:00 AEL (9.8 ± 0.4 in wild type, 4.9 ± 0.3 in *Cdc42*^*−/−*^, p < 0.0001; Student’s *t*-test) ^51^, highlighting the critical role of Cdc42 in the dendritogenesis process of aCC motoneurons.

The role of *Cdc42* in regulating cytoskeletal dynamics for morphogenesis depends on its activation, which allows it to bind to downstream effectors ^59, 60^. To investigate the mechanisms underlying Cdc42 activation, we began by screening RNA interference (RNAi) constructs targeting 18 genes that encode Cdc42 GEFs (**Extended Data** Fig. 1b). We expressed *UAS-RNAi* constructs in aCC motoneurons using an *even-skipped-GAL4* (*eve-GAL4*) driver line ^70^. The expression of the *GAL4* transgene was initiated four hours prior to the onset of dendritogenesis and continued throughout embryogenesis. This RNAi approach has proven to be exceptionally powerful, as we have previously identified numerous genes crucial for aCC morphogenesis ^51, 52^. Among the 18 genes screened, knocking down any one of the genes encoding Vav and RhoGEF2 resulted in a severe reduction in the number of dendritic filopodia (**Extended Data** Fig. 1b). To further narrow down the gene involved in dendritogenesis, we analyzed embryos homozygous for amorphic alleles of Vav or RhoGEF2 (*Vav*^*−/−*^ or *RhoGEF2*^*−/−*^). Among these, *Vav*^*−/−*^ embryos exhibited the most pronounced reduction in the number of dendritic filopodia (Fig. 1c **and Extended Data** Fig. 1c). This led us to focus on *Vav*, which encodes a cytosolic protein from the Dbl GEF superfamily. Unlike vertebrates, which possess three *VAV* genes, *Drosophila* has only one *Vav* gene ^71, 72^. Previous studies have shown that *Vav* is expressed early during embryogenesis and plays a critical role in axonal growth within a subset of interneurons. However, in contrast to these interneurons, aCC motoneuron axon development occurs normally in *Vav* ^*−/−*^ mutants, while dendritogenesis is severely impaired. Notably, the loss of dendritic filopodia in *Vav* ^*−/−*^ mutants was rescued by reintroducing *Vav* into aCC motoneurons (Fig. 1c). Together, these results demonstrate that *Vav* is a key GEF that functions in a cell-autonomous manner within aCC motoneurons to promote dendritic filopodia formation.

### Cdc42 GTPase is Activated by Vav in the aCC Motoneuron

**Figure 2:**
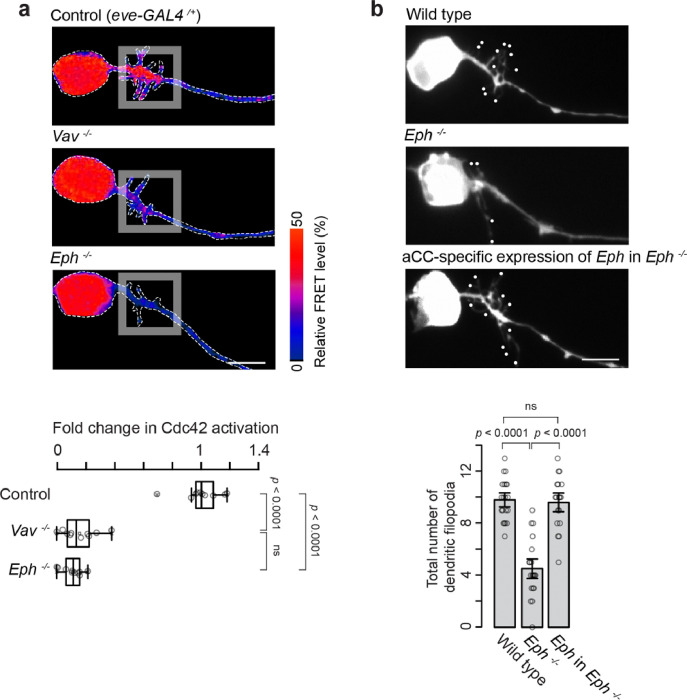
Vav and Eph Regulate Cdc42 Activation and Dendritic Filopodia Outgrowth **(a**) Pseudocolor images of aCC motoneurons in control (*eve-GAL4*^*/+*^), *Vav*^−/−^, and *Eph*^−*/*−^ embryos at 15:00 AEL, showing aProbe FRET signals, with warmer colors indicating higher Cdc42 activation levels. Dashed lines outline individual neurons. Quantification of Cdc42 activation in dendritic filopodia regions is shown below. Statistical analysis was performed by one-way ANOVA (*F*_*2,27*_ = 204, *p* = 0.0 × e^0^) with Tukey’s post hoc tests (*n* = 10 neurons per genotype). Analyses of Cdc42 activation in all other figures are also shown at 15:00 AEL. (**b**) Representative images of dendritic filopodia outgrowth in wild-type, *Eph*^−*/*−^, and aCC-specific expression of *Eph* in *Eph*^−*/*−^ embryos. Quantification of dendritic filopodia number is shown below. Statistical analysis was performed by one-way ANOVA (*F*_*2,55*_ = 45, *p* = 2.1 × e^−12^) with Tukey’s post hoc tests (*n* = 18–20 neurons per genotype). Scale bars: 5 mm.

To investigate whether the observed reduction in dendritic filopodia in *Vav* ^*−/−*^ mutants is a result of decreased Cdc42 activation, we imaged Cdc42 activity in aCC motoneurons. For this purpose, we previously generated transgenic flies expressing a fluorescence resonance energy transfer (FRET)-based Cdc42 activation probe, referred to as aProbe (**Extended Data** Fig. 2a) ^51^. When driven by the *eve-GAL4* driver, this probe revealed the presence of a FRET signal in the cell bodies throughout development, consistent with the understanding that *Cdc42* has multiple roles in maintaining cellular homeostasis ^73–75^. In addition, we detected a FRET signal in the proximal region of the axon. To further quantify changes in the FRET signal over time, we measured the region where the axon shaft spans from 5 to 30 μm from the midline (**Extended Data** Fig. 2b). Our analysis of the FRET signals revealed the dynamic nature of Cdc42 activation: the activation is absent before 13:00 AEL but begins to be consistently observed between 13:00 and 15:00 AEL (**Extended Data** Fig. 2c). This timing aligns with the emergence of filopodia. Furthermore, to assess the role of *Vav* in Cdc42 activation in the proximal axon region, we compared FRET signals in control flies to those in *Vav* ^*−/−*^ mutants at the critical time point of 15:00 AEL. We observed a significant reduction in Cdc42 activation within this region in *Vav* ^*−/−*^ mutants (Fig. 2a). Notably, Cdc42 activation remains normal in the cell body, suggesting that GEFs other than Vav regulate Cdc42 in this area. These findings provide strong evidence that Vav specifically regulates Cdc42 activation at the site of dendritogenesis.

### The Eph Receptor Activates Cdc42 and Plays a Cell-Autonomous Role in Dendritogenesis

Given the consistent and temporally precise regulation of Cdc42 activation across embryos, we hypothesized that this regulation might depend on extracellular signaling molecules engaging cell surface receptors that act upstream of Vav signaling. In a previous RNAi screen of 20 cell surface receptors, we focused on their roles in dendritic filopodia formation in aCC motoneurons. Using the *eve-GAL4* driver to express *UAS-RNAi* constructs, we identified several receptors whose knockdown significantly reduced the number of dendritic filopodia. Notably, the Down syndrome cell adhesion molecule (Dscam1) and the Eph receptor tyrosine kinase emerged as key regulators in this process. While previous studies suggested the involvement of *Dscam1* in aCC dendritogenesis, our observations revealed that *Dscam1* is not linked to Cdc42 activation, as normal activation occurred in *Dscam1* null mutants ^52^. This finding shifted our focus to the Eph receptor tyrosine kinase as a potential key regulator of this pathway. To investigate this further, we examined Cdc42 activation patterns in *Eph*^*x652*^ null mutants (*Eph* ^*−/−*^). The genetic simplicity of *Drosophila*, which contains only a single *Eph* gene ^76^, allowed us to directly assess the impact of *Eph* disruption on Cdc42 activation. Our results confirmed our hypothesis: in *Eph*^*−/−*^ mutants, we observed an almost complete absence of Cdc42 activation in the proximal axon (Fig. 2a). This strongly indicates that the Eph receptor plays a critical role in regulating Cdc42 activation during dendritogenesis.

To further validate our findings, we assessed the number of dendritic filopodia in *Eph*^*−/−*^ mutants, providing a critical validation experiment for our RNAi results. We observed a significant reduction in dendritic filopodia in *Eph* ^*−/−*^ embryos (Fig. 2b), with this decrease comparable to that observed with *Eph* RNAi expression in aCC motoneurons using the *eve-GAL4* driver (5.5 ± 0.5 μm in knockout, 4.1 ± 0.7 μm in knockdown, *p* = 0.14; Welch’s *t*-test). Importantly, this reduction in dendritic filopodia was successfully rescued by expressing *Eph* specifically in aCC motoneurons (Fig. 2b). These results suggest that the Eph receptor functions in a cell-autonomous manner to regulate Cdc42 activation and promote aCC dendritogenesis.

### The SH2 Domain of Vav Interacts with the Juxtamembrane Tyrosine Residues of Eph

**Figure 3:**
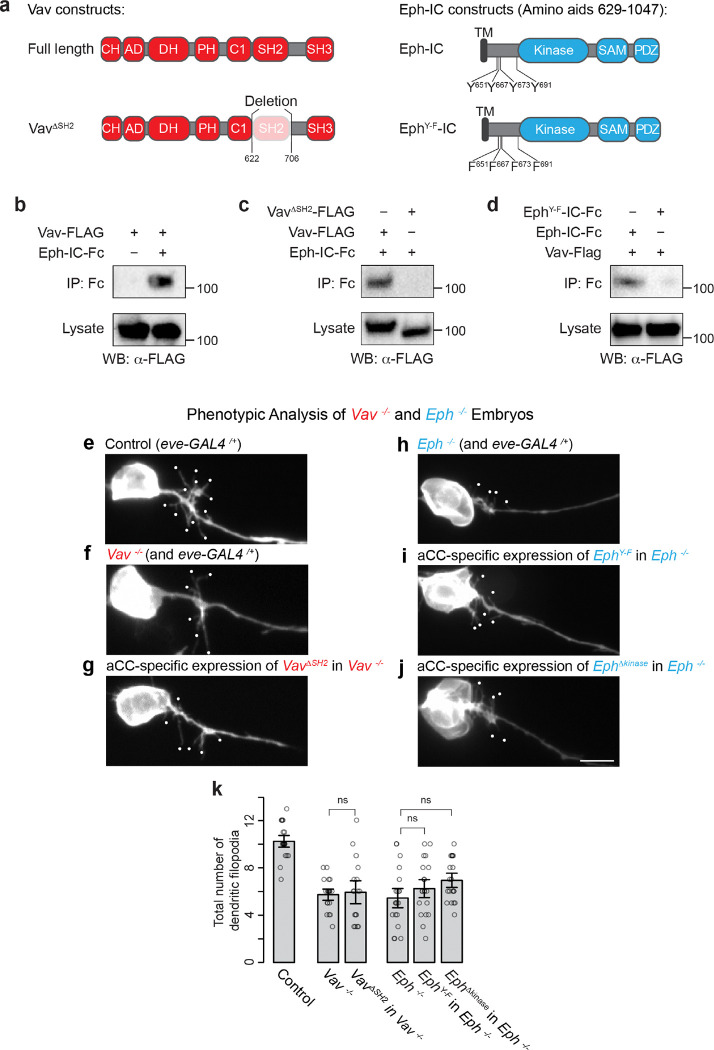
Essential Roles of Vav SH2 Domain and Eph Tyrosine Residues in Dendritic Outgrowth **(a**) Schematic of Vav and Eph constructs used in co-IP assays, indicating amino acid positions. TM, transmembrane domain; IC, intracellular domain. (**b**-**d**) Western blots showing co-IP results between Vav-FLAG and Eph-Fc. “IP” denotes immunoprecipitation, and “WB” indicates Western blotting. Similar results were obtained from three independent experiments. (**e**-**j**) Representative images of dendritic outgrowth under various conditions: control, *Vav*^−*/*−^, aCC-specific expression of *Vav*^*ΔSH2*^ in *Vav*^−*/*−^, *Eph*^−*/*−^, aCC-specific expression of *Eph*^*Y-F*^, and *Eph*^*Δkinase*^ in *Eph*^−*/*−^ embryos. (**k**) Quantification of dendritic filopodia per aCC neuron for the indicated genotypes. Statistical analysis was performed using Welch’s *t*-test and one-way ANOVA (*F*_*2,57*_ = 2.4, *p* = 0.1) with Tukey’s post hoc tests (*n* = 18–20 neurons per genotype). Scale bars: 5 mm.

Thus far, we explore the activation of Cdc42, focusing on the roles of Vav and Eph. While the activation of Cdc42 by Vav and Eph appears to be a novel finding, there is a precedent in mammalian systems where EphA4 and Vav2 have been shown to interact and form a complex that activates Rac1, another small GTPase ^77^. This observation led us to hypothesize that *Drosophila* Vav and Eph might interact in a similar manner, but with the specific outcome of activating Cdc42. To investigate this hypothesis, we conducted biochemical assays using the *Drosophila* S2 cell line. We transiently transfected these cells with expression plasmids encoding FLAG-tagged Vav (FLAG-Vav, a construct where the FLAG epitope is fused to *Drosophila* Vav) and Eph-IC-Fc (the intracellular domain of *Drosophila* Eph fused to the human Fc domain for protein A/G bead interaction) (Fig. 3a). After transfection, we performed immunoprecipitation to isolate the Eph receptors from the cell lysates. The immunoprecipitated proteins were then analyzed via Western blotting to detect potential interactions between Vav and Eph.

Our initial experiment successfully demonstrated the interaction between Vav and Eph (Fig. 3b). Building on this result, we next sought to identify the specific regions of interaction between the two proteins. Given the cross-species conservation of Eph-Vav interactions, we hypothesized that the SH2 domain of Vav binds to phosphorylated tyrosine residues on Eph. To test this, we first examined the role of the SH2 domain in Vav. We generated a Vav deletion mutant lacking the SH2 domain (Vav^ΔSH2^) and found that this mutant did not interact with Eph (Fig. 3c), underscoring the essential role of the SH2 domain in mediating the interaction. SH2 domains typically bind preferentially to phosphorylated tyrosine residues. Thus, we shifted our focus to the tyrosine residues of Eph, specifically in the juxtamembrane (JM) region, which is known to be phosphorylated by the kinase activity of mouse EphA4. In *Drosophila* Eph, there are four tyrosine residues in this region. When we substituted these JM tyrosines with phenylalanines (Y651F/Y667F/Y673F/Y691F), we observed that Vav did not interact with the *Eph* mutant (Fig. 3d). In summary, our findings from *Drosophila* S2 cell assays reveal a specific interaction between the JM tyrosines of Eph and the SH2 domain of Vav.

### The Eph-Vav Interaction Promotes Dendritic Filopodia Outgrowth Following Eph Autophosphorylation

To further investigate the physiological relevance of the interaction between Vav and Eph, we tested the deletion and mutation constructs *in vivo*. Based on the successful rescue of the loss-of-dendrite phenotype in aCC motoneurons observed with wild-type constructs (Figs. 1c and 2b), we hypothesized that if the interaction sites are essential, these mutant constructs would fail to rescue the phenotype.

First, we specifically expressed *Vav*^*ΔSH2*^—the deletion mutant lacking the SH2 domain—in aCC neurons of *Vav*^*−/−*^ mutants. The expression of *Vav*^*ΔSH2*^ failed to rescue the dendritic phenotype (Figs. 3e–g and 3k). Next, we expressed *Eph*^*Y−F*^—a mutant that prevents phosphorylation—in *Eph*^*−/−*^ mutants. Similar to *Vav*^*ΔSH2*^, *Eph*^*Y−F*^ expression also failed to rescue the phenotype (Figs. 3h–i and 3k). These results demonstrate that the phosphorylation-dependent interaction between Vav and Eph is crucial for aCC dendritogenesis.

Since Eph phosphorylation typically depends on its intrinsic tyrosine kinase activity ^78, 79^, we further examined the role of the intracellular tyrosine kinase domain. We generated an Eph deletion mutant lacking this domain (*Eph*^*Δkinase*^) and expressed it in *Eph*^*−/−*^ mutants. Expression of *Eph*^*Δkinase*^ also failed to rescue the phenotype (Figs. 3j–k). Collectively, our data suggest that the kinase domain of Eph autophosphorylates its juxtamembrane tyrosines, creating docking sites for the SH2 domain of Vav. This phosphorylation event leads to the localization of Vav to the plasma membrane.

### Membrane Localization of Vav Is Essential for Dendritic Filopodia Outgrowth

This membrane localization of Vav is potentially crucial for the activation of Cdc42, as Cdc42 typically localizes to the membrane via its CAAX motif. Consequently, Vav would have a higher likelihood of interacting with Cdc42 if it is stably localized on the membrane. To test this hypothesis, we engineered a membrane-anchored version of Vav (myr-Vav^DH−PH^) by fusing a myristoylation signal to its RhoGEF domain, thereby mimicking the membrane localization of Vav. We then investigated whether this modification could compensate for the absence of Eph. To do so, we expressed the *myr-Vav*^*DH−PH*^ construct in *Eph*^*−/−*^ embryos using the *eve-GAL4* driver. Given the ongoing activity of endogenous Vav, we aimed to maximize the expression of *myr-Vav*^*DH−PH*^ by using two copies of the *GAL4* driver. Remarkably, overexpressing *myr-Vav*^*DH−PH*^ successfully rescued the *Eph* mutant phenotype (**Extended Data** Figs. 3a-c and 3f). To confirm that this rescue effect was specifically due to the membrane localization of Vav, and not merely an increase in its overall levels, we overexpressed wild-type *Vav*. However, this did not yield a similar rescue (**Extended Data** Figs. 3d and 3f). These findings underscore the critical role of Vav’s plasma membrane localization, facilitated by Eph, in driving proper aCC dendritogenesis.

Furthermore, we investigated the necessity of Vav’s GEF catalytic activity in this rescue experiment. To do this, we introduced a double-point mutation (L356A, K357A) into the DH domain, which is known to abolish GEF catalytic activity ^80^. Significantly, overexpression of this catalytically inactive form (*myr-vav*^*DH[AA]−PH*^) failed to rescue the *Eph* mutant phenotype (**Extended Data** Figs. 3e and 3f). These results strongly suggest that Vav’s GEF activity is essential for its role in aCC dendritogenesis.

### Vap33 binds to Eph to Activate Cdc42 During aCC Dendritogenesis

**Figure 4:**
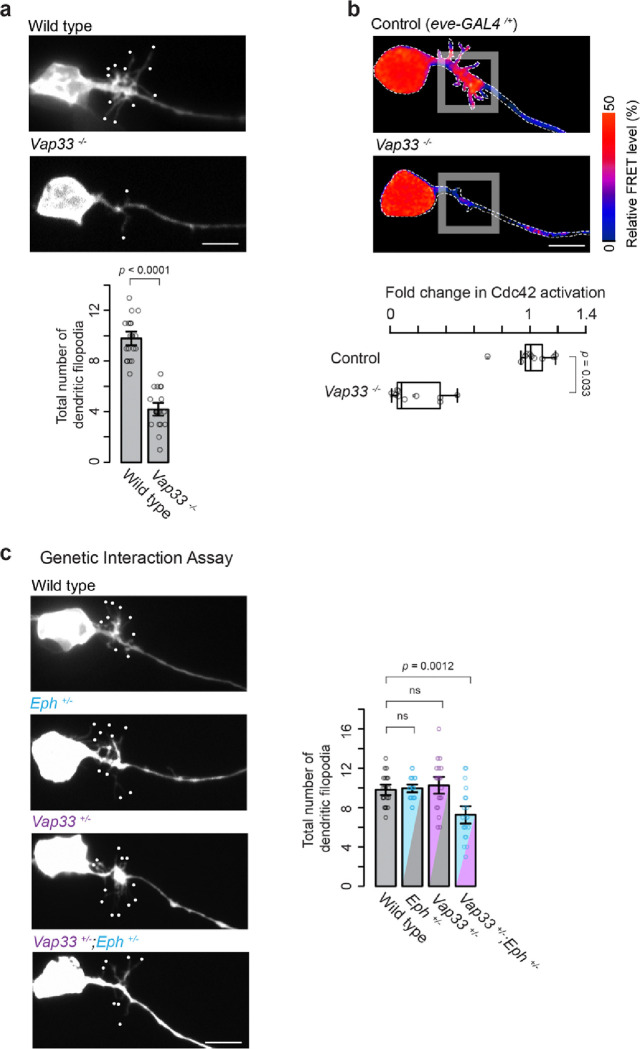
Vap33 Promotes Dendritogenesis and Activates Cdc42 via Interaction with Eph Receptors **(a**) Representative images of dendritic outgrowth in wild-type and *Vap33*^−*/*−^ embryos. Statistical analysis (two-sample *t*-test) shows significant differences (*n* = 20 neurons per genotype). (**b**) Pseudocolor images showing aProbe responses in control and *Vap33*^−*/*−^ embryos. Quantification of Cdc42 activation levels per aCC neuron indicates significant differences (Mann-Whitney *U* test, *n* =10 neurons per genotype). (**c**) Images of dendritic outgrowth in embryos heterozygous for *Eph*, *Vap33*, and double heterozygous for both. Dendritic filopodia counts per aCC neuron reveal significant differences (one-way ANOVA, *F*_*3,76*_ = 8.9, *p* = 3.8 × e^−5^) with Tukey’s post hoc tests (*n* = 20 neurons per genotype). Scale bars: 5 mm.

It appears that Eph phosphorylation is a key event in the downstream signaling of Eph. The next question is how Eph becomes phosphorylated. Typically, phosphorylation of Eph receptors is triggered by binding to specific ligands, which in turn dimerize the receptors. In *Drosophila*, *Ephrin* and *Vap33* have been identified as the primary ligands for Eph receptors ^76, 81, 82^. We sought to determine the role of these ligands in dendritic filopodia formation within aCC motoneurons. To explore this, we examined *Ephrin*^*I95*^ and *Vap33*^*Δ31*^ mutant embryos, which lack their respective proteins. The results of this analysis were quite revealing. We observed a significant reduction in the number of dendritic filopodia in *Vap33*^*−/−*^ embryos (Fig. 4a), whereas *Ephrin*^*−/−*^ embryos did not show a substantial difference from wild-type embryos (**Extended Data** Fig. 4a). Importantly, this reduction in filopodia was not due to global changes in the circuit architecture of the VNC, as the neuropiles remained intact (**Extended Data 4b**).

This distinct phenotype in *Vap33*^*−/−*^ mutants has directed our research focus towards understanding Vap33’s function. To begin, we assessed whether the *Vap33* mutation influences Cdc42 activation. Our analysis revealed that Cdc42 activation is significantly decreased in *Vap33*^*−/−*^ embryos (Fig. 4b). Notably, the extent of this reduction in Cdc42 activation is comparable to what we observed in *Vav*^*−/−*^ or *Eph*^*−/−*^ embryos. Importantly, none of these mutations, including *Vap33*^*−/−*^, affect Cdc42 activation in the cell body (see also Fig. 2a), suggesting that Cdc42 activation is regulated by distinct signaling pathways in the cell body versus the axons.

Bellen’s lab previously demonstrated that Vap33 interacts with Eph using a co-immunoprecipitation (co-IP) assay ^81^. Building on this, we now show that Vap33 and Eph both activate Cdc42 at the same region of the aCC axon shaft (Figs. 2a and 4b), suggesting they may function together in this process. To directly assess whether they are part of the same signaling pathway in aCC dendritogenesis, we examined their genetic interaction. Our initial attempt to generate a double homozygous combination was unsuccessful due to the limited availability of genetic tools necessary to combine the *Vap33* and *Eph* genes, located on the X and 4th chromosomes respectively, within the same animals. As an alternative, we tested a double heterozygous combination. Remarkably, these double heterozygous mutants exhibited a subtle but statistically significant reduction in dendritic filopodia formation in aCC motoneurons (Fig. 4c), a reduction not observed in either of the single heterozygous mutants (Fig. 4c). This synergistic effect in the double heterozygous mutants suggests that the interaction between Vap33 and Eph contributes to the outgrowth of dendritic filopodia in aCC motoneurons.

### aCC Dendritic Filopodia Outgrowth Requires the Non-Cell-Autonomous Role of Vap33

**Figure 5:**
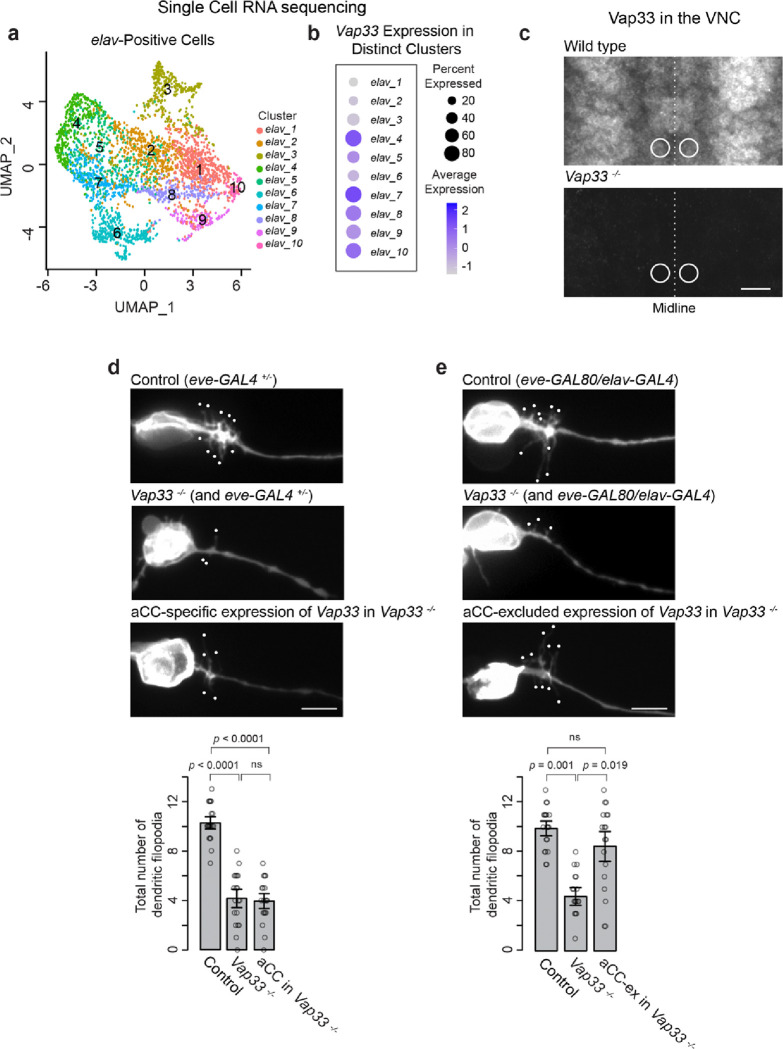
Extracellular Vap33 Is Required for aCC Dendritogenesis (**a**) UMAP analysis of embryos (7:20–11:20 AEL) showing *elav*-positive neuronal cells forming 10 clusters, color-coded by defined subsets. (**b**) Dot plot from scRNA-seq data showing *Vap33* expression across the 10 clusters (data from Seroka et al.). (**c**) Anti-Vap33 staining in single ventral nerve cord (VNC) segments of wild-type and *Vap33*^−*/*−^ embryos at 11:00 AEL. Circles indicate aCC motoneuron cell bodies. (**d**, **e**) Images of dendritic filopodia in control, *Vap33*^−*/*−^, and *Vap33*^−*/*−^ embryos with aCC-specific or aCC-excluded expression of *Vap33*. Quantification of dendritic filopodia per aCC neuron reveals significant differences (one-way ANOVA, *F*_*2,55*_ = 76, *p* = 1.1 × e^−16^) with Tukey’s post hoc tests (*n* = 18–19 neurons per genotype). Scale bars: 10 mm in (c); 5 mm in (d, e).

Next, we sought to characterize when and where Vap33 interacts with Eph. Two possible scenarios were considered: (1) Vap33 could be expressed within the aCC motoneuron itself, binding to Eph receptors either within the secretory pathway or at the plasma membrane, or (2) Vap33 could be expressed in nearby neurons, exerting its effects on aCC motoneurons through non-cell-autonomous mechanisms. To investigate this, we first analyzed which neurons express Vap33 using recent single-cell RNA sequencing data ^83^. By 11:20 AEL (1.5 hours before dendritogenesis begins), Vap33 is expressed in specific subsets of neurons (Fig. 5a–b). To further confirm this, we performed immunostaining on wild-type embryos using available Vap33-specific antibodies. Our observations revealed that Vap33 is present in many, but not all, neurons within the VNC, including aCC, as early as 11:00 AEL (Fig. 5c). However, these results do not definitively support either of the proposed scenarios. To pinpoint the specific location of its source, we conducted a series of rescue experiments in *Vap33* ^*−/−*^ mutants. We first expressed *Vap33* specifically in aCC motoneurons. This aCC-specific expression did not rescue the mutant phenotype (Fig. 5d). In contrast, when we expressed Vap33 in all neurons except aCC using the pan-neuronal *elav-GAL4* driver combined with the *eve-GAL80* repressor, we successfully rescued the phenotype (Fig. 5e). These results strongly support the idea that Vap33 is produced externally to aCC motoneurons and acts as an Eph ligand in a non-cell-autonomous manner to regulate dendritic filopodia outgrowth.

### Vap33 is a Temporal Regulator for Dendritic Filopodia Formation in Motoneurons

**Figure 6:**
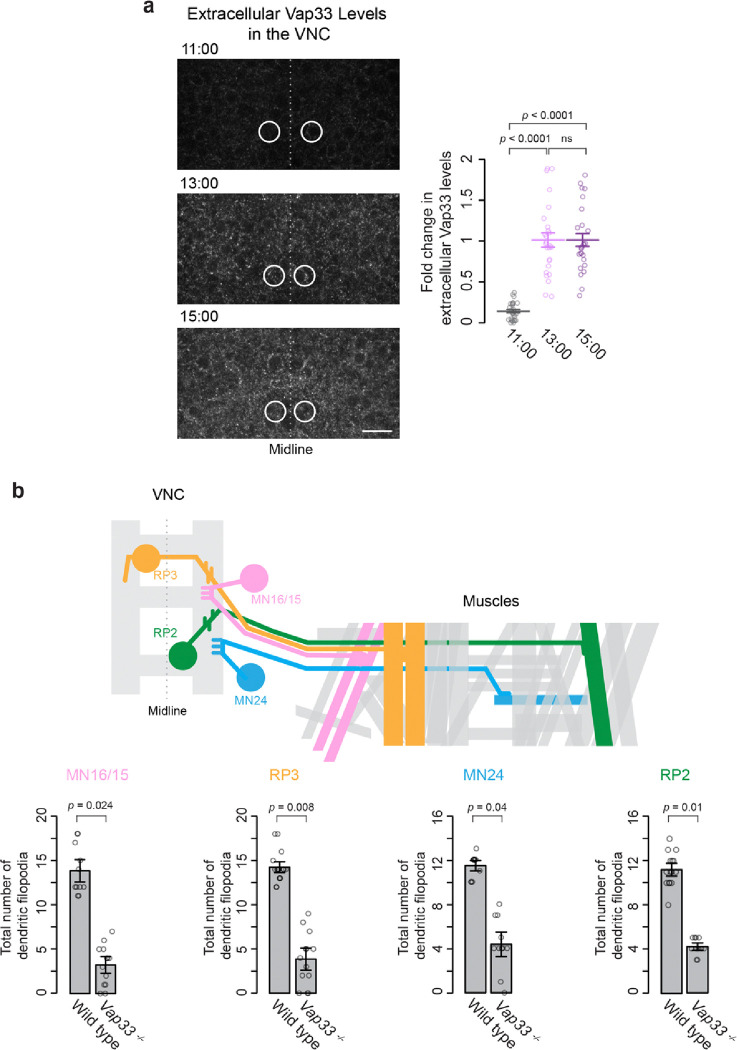
Extracellular Vap33 Influences Global Dendritogenesis of Motoneurons (**a**) Anti-Vap33 staining in wild-type embryos at three time points (11:00, 13:00, and 15:00 AEL), showing detection of extracellular Vap33 without detergent treatment. Circles indicate aCC motoneuron cell bodies. Quantification of extracellular Vap33 levels normalized to *Vap33*^−*/*−^ mutants and wild-type embryos at 15:00 AEL (set as 0 and 1, respectively). Statistical analysis was performed using one-way ANOVA (*F*_*2,67*_ = 56, *p* = 1.1 × e^−15^) with Tukey’s post hoc tests (*n* = 26–27 segments per time point). (**b**) Top: Schematic of four motoneurons (MN16/15, RP3, MN24, PR2) in a single embryonic VNC segment, each projecting to specific target muscles. Bottom: Statistical analysis (Mann-Whitney *U* test) shows significant differences in dendritogenesis between motoneurons of wild-type and *Vap33*^−*/*−^ embryos (*n* = 11–17 neurons per genotype). Scale bar: 10 mm.

Our final question centers on how the timing of dendritogenesis is determined. Importantly, Vap33 is already expressed by 11:00 AEL (Figs. 5a–b), well before the onset of dendritogenesis. Similarly, Eph, Vav, and Cdc42 are all known to be expressed in the VNC by at least 11:00 AEL ^51, 71, 76^. Thus, the necessary components for dendritogenesis are present early in development. The precise timing may be controlled by regulatory events downstream of protein expression. One intriguing possibility is that Vap33 may start being secreted externally at the onset of dendritogenesis, around 13:00 AEL. In other words, the availability of Vap33 as an Eph ligand could serve as a time-determining factor for dendritogenesis. To test this hypothesis, we performed immunostaining on wild-type embryos without the use of detergents. Unlike typical immunofluorescence, which includes a permeabilization step to detect both intracellular and extracellular components (Fig. 5c), this method identifies only the extracellular portion of Vap33. The effectiveness of this approach has been demonstrated in a previous study by Tsuda et al. ^81^. Using this method, we observed that in the VNC, the fluorescence level of Vap33 at 11:00 AEL is undetectable (Fig. 6a), resembling the signal seen in *Vap33* ^*−/−*^ embryos at 15:00 AEL, which serves as a negative control. Intriguingly, the fluorescence level begins to increase substantially at 13:00 AEL (Fig. 6a), coinciding with the onset of dendritogenesis. This observation strongly supports our hypothesis that Vap33 secretion acts as a timing signal for dendritogenesis.

Furthermore, we observed that the extracellular portion of Vap33 is uniformly distributed across both the anteroposterior and mediolateral axes of the VNC, contrasting with the more localized pattern of total Vap33 distribution (**Extended Data** Fig. 5a). This broad extracellular distribution suggests that secreted Vap33 is not restricted to aCC motoneurons but is likely used by other motoneurons, most of which extend their dendrites within the same dorsal region of the VNC as aCC ^84^. In *Drosophila* embryos, each hemisegment contains approximately 36 motoneurons, with each typically innervating one of the 30 somatic muscles. During development, these axons exit the VNC via one of five main nerve branches: intersegmental nerve (ISN), ISNb, ISNd, SNa, and SNc. Each motoneuron, depending on its nerve branch, projects its dendrites to specific anteroposterior and mediolateral territories within the VNC ^44, 85^. To explore this further, we selected four motoneurons from distinct nerve branches with varying birth times and locations: motoneuron 16/15 (MN16/15), raw prawn 3 (RP3), MN24, and RP2 (Fig. 6b). Our observations revealed that dendritic filopodia for these motoneurons begin to emerge at approximately 13:00 AEL and become stabilized by 15:00 AEL (data not shown). In *Vap33* ^−/−^ embryos, these motoneurons exhibited a similar degree of dendrite loss as aCC motoneurons at 15:00 AEL (Fig. 6b and **Extended Data** Fig. 5b). Taken together, these findings indicate that Vap33 secretion plays a crucial role in the temporally coordinated outgrowth of dendritic filopodia, contributing to the development of the myotopic map and ultimately influencing the formation of the locomotion circuit.

## Discussion

**Figure 7:**
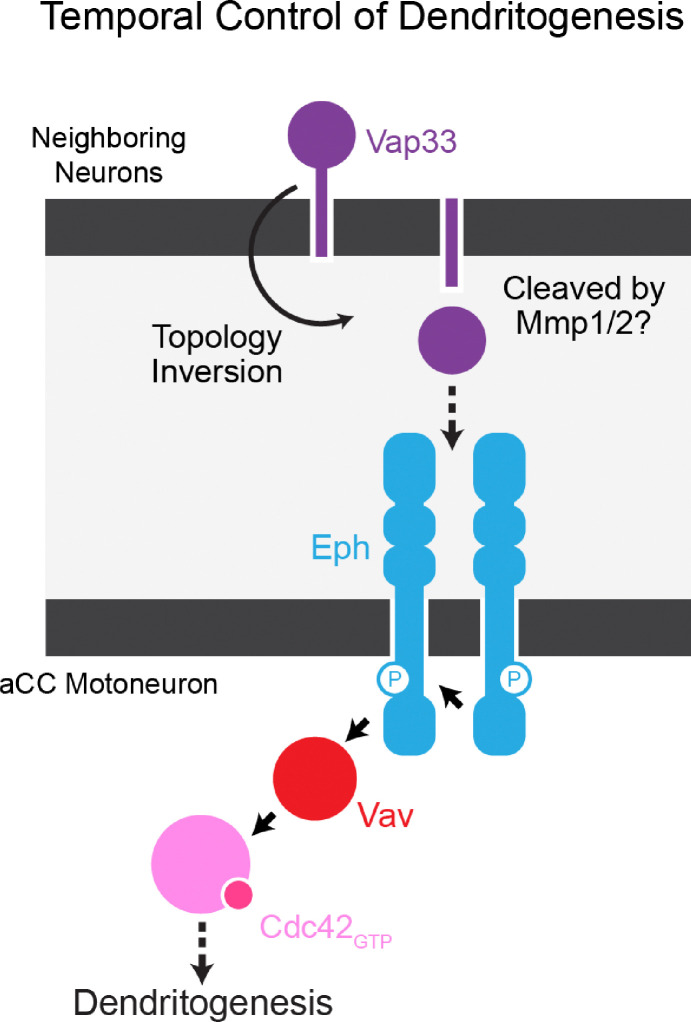
Model of Temporal Regulation of Dendritogenesis via Cdc42 Activation Schematic model illustrating how Vap33, Eph, and Vav coordinate to temporally regulate dendritogenesis through activation of Cdc42 in motoneurons. This pathway controls the precise timing of dendritic filopodia formation during development.

Here, we identify a novel signaling cascade that temporally activates Cdc42 (Fig. 7). At the onset of dendritogenesis, Vap33 is exposed to the extracellular space, enabling it to bind to Eph receptors. This Vap33-Eph interaction induces the membrane localization of Vav, which in turn leads to the timely activation of Cdc42. Notably, recent evidence suggests that dysfunction of VAPB, the human ortholog of Vap33, is linked to the progression of ALS8 and SMA pathologies (see further discussion below). Our findings provide new insights into how VAPB signaling influences the formation of motor circuitry, shedding light on its role in both normal development and disease processes in humans.

In this study, we reveal a previously uncharacterized function of the Rho family guanine nucleotide exchange factor (GEF) Vav in *Drosophila*. Our findings suggest that Vav serves as a crucial molecular hub, linking Eph receptor activity to Cdc42-dependent dendritic outgrowth. The key evidence supporting this linkage includes: (1) Disruption of either Eph or Vav function leads to similar dendritic outgrowth defects (Figs. 1 and 2). (2) Mutations in either Vav or Eph result in abolished Cdc42 activation (Fig. 2). (3) Vav interacts directly with the Eph receptor (Fig. 3). (4) The interaction domains within Vav and Eph are essential for their role in dendritic development (Fig. 3). (5) Expression of a membrane-tethered version of Vav rescues dendritic growth defects in Eph mutants, while a version of Vav lacking GEF activity does not (**Extended Data** Fig. 3). These observations suggest a defined signaling cascade: Eph receptor activation initiates a critical process by autophosphorylating its tyrosine-rich juxtamembrane region. This phosphorylation enables the SH2 domain of Vav to bind to the phosphorylated tyrosines, facilitating the recruitment of Vav to the plasma membrane. Membrane localization of Vav is essential for activating Cdc42, which likely drives actin polymerization, promoting the protrusion of dendritic filopodia necessary for dendritic outgrowth.

Eph-Vav signaling, initially discovered in the context of axon guidance in mouse retinal ganglion cells (RGCs), highlights the pivotal role of Vav2 in activating Rac1 ^77^. This Rac1 activation is crucial for growth cone repulsion, affecting both plasma membrane internalization and actin cytoskeleton contractility. In *Drosophila*, the genome encodes three Rac genes (*Rac1*, *Rac2*, and *Mtl*) and a single Cdc42 gene ^86–88^. Given the high amino acid similarity among these GTPases, it is plausible that all could be activated by Vav. This raises a critical question: How does Vav selectively activate Cdc42, or other GTPases? A potential explanation involves the developmental regulation of gene expression. Our analysis of available scRNA-seq data reveals differential expressions of these GTPases in aCC motoneurons (**Extended Data** Fig. 6a). Between 10:20 and 11:20 AEL, *Cdc42* is expressed at levels similar to *Rac1*, while *Rac2* and *Mtl* are less abundant. During this developmental window, Vav may activate both Cdc42 and Rac1. However, it is noteworthy that our previous findings showed no defects in aCC dendritogenesis in *Rac* triple mutants, though axon guidance was affected in these mutants ^52, 89^. These observations suggest an additional layer of regulation for Vav, potentially activating Cdc42 or Rac1 in different subcellular locations, thereby leading to distinct downstream signaling pathways. This spatial regulation could result in specific patterns of actin cytoskeletal rearrangements, influencing neuronal morphology in diverse ways.

Vap33 has primarily been characterized as an ER-resident protein in *Drosophila*, involved in membrane trafficking, lipid transfer, and forming membrane contact sites between the ER and other organelles ^81,^ 90–93. It has also been shown to modulate microtubule reorganization to maintain synaptic physiology in larval neuromuscular junctions ^94, 95^. Notably, VAPB, which is the human ortholog of Vap33, is linked to neurodegenerative diseases such as amyotrophic lateral sclerosis type 8 (ALS8) and has been the subject of intensive investigation ^66–68^. VAPB is characterized by an MSP domain, a coiled-coil domain, and a transmembrane domain, lacking an N-terminal signal peptide ^96, 97^. This structural configuration directs a large portion of the protein into the cytoplasm on the ER membrane, classifying it as a type IV transmembrane protein. Unlike type I transmembrane proteins that face the ER lumen, it has long been unclear how VAPB is transported from the ER to the extracellular space ^81, 98^. Our recent studies in cultured *Drosophila* S2 cells have begun to shed light on the mechanisms behind this transport ^99^. We demonstrated that Vap33 is transported to the plasma membrane and then undergoes a “topology inversion,” where its cytoplasmic portion is reoriented to the extracellular space. Following this inversion, Vap33 is cleaved by Matrix metalloproteinases 1/2 (Mmp 1/2), releasing the MSP domain. We hypothesize that this topology inversion of Vap33 occurs *in vivo* as well (Fig. 7). In our model, subsets of neurons secrete Vap33 extracellularly at the onset of dendritogenesis. The MSP domain then interacts with Eph receptors on the plasma membranes of aCC motoneurons, triggering the timely formation of dendritic filopodia. However, key questions remain: Does Vap33 remain on the plasma membrane after secretion, or is it cleaved before interacting with Eph on aCC motoneurons? If cleavage occurs, how do Mmp 1/2 facilitate this process? These questions are critical to understanding the full mechanism of Vap33 function in this context.

Similar to *Drosophila*, mammalian motoneurons of specific subclasses extend their dendrites into distinct territories within the spinal cord ^100–102^. However, the molecular mechanisms controlling the spatiotemporal formation of motor dendrites are yet to be determined. Intriguingly, emerging research on motor neuron diseases such as ALS and SMA provides a potential link to our study. These patients are characterized by progressive muscle weakness and atrophy from progressive degeneration of motoneurons ^103, 104^. Linkage analysis and subsequent Sanger sequencing of candidate genes in these patients revealed mutations in the VAPB gene ^105–108^. One variant involves the substitution of proline with serine at amino acid position 56 (VAPB^P56S^). Overexpression of VAPB^P56S^ in cultured mammalian cells leads to its aggregation at the ER, trapping endogenous VAP within these mutant aggregates, which suggests a dominant negative function of this variant ^109–111^. Consequently, this overexpression may reduce the secretion of endogenous VAPB ^112^. Furthermore, even when VAPB^P56S^ is exposed extracellularly, it shows resistance to proteolysis, implying that it would not be cleaved ^113, 114^.

Consistent with this, over 50% of sporadic ALS patients either lack or have significantly reduced levels of the MSP fragment in their cerebrospinal fluid ^115^. These findings suggest that abnormalities in VAPB secretion and cleavage are associated with the development of motor neuron disorders. Based on our results, exploring its extracellular regulation could provide new insights into the formation of the myotopic map in the spinal cord and provide a deeper understanding of the etiology of these motor neuron disorders.

We convincingly demonstrate that Cdc42 is activated via Vap33-Eph-Vav signaling at the onset of dendritogenesis. However, we do not address why Cdc42 activation is spatially restricted to the proximal region of axons (e.g., **Extended Data** Fig. 2c). The mechanisms underlying this spatial confinement remain poorly understood. Since secreted Vap33 is uniformly distributed in the VNC (**Extended Data** Fig. 5a), it is likely that this spatial regulation is independent of Vap33. We hypothesize that the localization of Eph receptors may play a critical role in determining the spatial restriction of Cdc42 activation. Previous research has shown that certain guidance receptors can localize to specific regions of axons, such as the proximal or distal segments, independently of direct cell-cell contact ^116^. For instance, the guidance receptor Derailed localizes to the proximal region of the longest neurite in single cultured *Drosophila* neurons. Building on these findings, we visualized the localization of Eph in cultured neurons and observed a similar proximal distribution in the longest neurites (**Extended Data** Fig. 6b). This suggests that neurons may have intrinsic mechanisms to direct Eph receptors to specific membrane compartments, which could spatially restrict the downstream activation of Cdc42. While this observation provides valuable insight, it remains to be determined whether this spatial organization is established and maintained *in vivo* and how it might be regulated.

## Methods

### Molecular Cloning

Complementary DNA sequences of *Vav* and *Eph* (LD25754 and RE61046) (*Drosophila* Gene Collection, BDGP) were PCR amplified and subcloned into *pACUH-superfolderGFP* (*sfGFP*) to generate C-terminal *sfGFP* fusions using the NotI restriction site. The *pACUH-sfGFP* vector includes the *sfGFP* sequence in *pACUH* (Addgene) at the *Not*I and *Xba*I sites. To create deletion constructs (*Vav*^*DSH2*^ and *Eph*^*Dkinase*^*)*, we performed site-directed mutagenesis using the Q5 Site-Directed Mutagenesis Kit (New England Biolabs). We deleted a DNA sequence encoding the SH2 domain of Vav (amino acids 622–706) or the kinase domain of Eph (amino acids 692–950) from *pACUH-Vav-sfGFP* or *pACUH-Eph-sfGFP*, respectively. To replace tyrosine (Y) with phenylalanine (F), we designed two pairs of mutagenesis primers. We performed two rounds of mutation reactions and introduced four Y-to-F substitutions to the juxtamembrane region of Eph. A synthesized DNA fragment (Integrated DNA Technologies) containing either *myr-Vav*^*GEF−PH*^ or *myr-Vav*^*GEF[AA]−PH*^ was cloned into the *Eco*RI and *Not*I sites of *pACUH-sfGFP*. Those Vav^GEF−PH^ constructs comprise a GEF-PH domain only (amino acids 220–541).

For co-immunoprecipitation assays, we designed plasmids to produce FLAG-tagged or Fc-tagged proteins. *Vav-3xFLAG* and *Vav*^*DSH2*^*-3xFLAG* fragments were PCR amplified from *pACUH-vav-sfGFP* and *pACUH-Vav*^*DSH2*^*-sfGFP*. Then, we introduced each fragment to the *pACUH* vector through the *Not*I and *Xba*I sites, constructing *pACUH-Vav-3xFLAG* and *pACUH-Vav*^*DSH2*^ *−3xFLAG*. Human *Fc* fragments were PCR amplified from the *pIB-Fc* vector (a gift from Thomas Kidd). *Eph-IC* and *Eph*^*Y−F*^*-IC* were generated via PCR from the full-length *Eph* and *Eph*^*Y−F*^. *Eph-IC-Fc* and *Eph*^*Y−F*^*-IC-Fc* were assembled into *Not*I- and *Xba*I-digested *pACUH*. *Eph-IC* encodes a fragment of the C-terminal region (amino acids 629–1047).

Standard subcloning, PCR amplification (PrimeSTAR Max polymerase, Takara Bio USA), and In-Fusion cloning (In-Fusion Snap Assembly Master Mix, Takara Bio USA) were used to generate all plasmids and intermediates. The oligonucleotides and gene fragments used are listed in **Supplementary Table 2**.

### Transgenic Line Production

After being sequenced, all plasmids were injected into *attP* embryos expressing *FC31* by Rainbow Transgenic Flies and Best Gene.

### Fly Stock

Standard *Drosophila* genetic techniques were used for all crosses. All flies were raised at 25°C. The following lines were obtained from the Bloomington *Drosophila* Stock Center: *eve-GAL4* (BDSC stock no. 7470 and 7473), *elav-GAL4* (8760), *UAS-sif RNAi* (61934), *UAS-Ziz RNAi* (54817), *UAS-RhoGEF3 RNAi* (42526), *UAS-Rtgef RNAi* (32947), *UAS-RhoGEF4 RNAi* (42550), *UAS-pbl RNAi* (36841), *UAS-Cdep RNAi* (31168), *UAS-PsGEF RNAi* (44061), *UAS-GEFmeso RNAi* (42545), *UAS-trio RNAi* (43549), *UAS-unc-89 RNAi* (34000), *UAS-RhoGEF64C RNAi* (31130), *UAS-Pura RNAi* (58270), *UAS-Zir RNAi* (53946), *UAS-spg RNAi* (35396), *UAS-Exn RNAi* (33373),*UAS-Vav RNAi* (39059), *UAS-RhoGEF2 RNAi* (34643), *UAS-Eph RNAi* (60006), and *UAS-FLAG-Vap33-HA* (39682). *Vav*^*2*^ and *Vav*^*3*^ mutants were provided by Dr. Marianne Malartre, The French National Centre for Scientific Research, France. *Vap33*^*Δ31*^ mutant was obtained from Drs. Hiroshi Tsuda and Hugo Bellen. The genotypes used in each experiment are listed in **Supplementary Table 1.**

### Primary Neuronal Culture

Flies were allowed to lay eggs on a grape agar plate overnight in a cage. The following day, the collected eggs were dechorionated in a 50% bleach solution for 5 minutes. After dechorionation, the eggs were gathered using a cell strainer (Corning) and gently transferred into SFX culture medium (Hyclone) in a new centrifuge tube using a pen brush. The embryos were mechanically dissociated by homogenization with a plastic homogenizer (Thermo Fisher Scientific), applying 10–20 strokes. To facilitate further dissociation, trypsin was added to the homogenate, and the samples were incubated at 37°C for 30 minutes. After incubation, 100 μL of fetal bovine serum (FBS; R&D Systems) was added to inhibit the trypsin activity. The mixture was centrifuged at 1,000 rpm for 2 minutes, and the supernatant was carefully removed. The resulting pellet was resuspended in SFX medium supplemented with penicillin/streptomycin (Corning), FBS, and insulin (MilliporeSigma). Finally, the cells were transferred into an 8-well chamber slide (Nunc Lab-Tek) for immunostaining.

### Immunohistochemistry

#### Dissected Embryos:

Dissected embryos were fixed with 4% paraformaldehyde in phosphate-buffered saline (PBS) for 10 minutes, washed in TBS (0.1% Triton X-100 in PBS), blocked in 10% bovine serum in TBS for 1 hour, and incubated with primary antibodies overnight at 4°C. The primary antibodies used included rabbit anti-Vap33 (a gift from Hiroshi Tsuda and Hugo Bellen; 1:10,000), mouse anti-Fasciclin II (Developmental Studies Hybridoma Bank; 1:100), and Alexa647-conjugated goat anti-HRP (Jackson ImmunoResearch Labs; 1:500). After washing, embryos were incubated with secondary antibodies conjugated to fluorescent dyes (Thermo Fisher Scientific; 1:500) for 2 hours at room temperature. Following extensive washes with TBS, embryos were mounted in 50% glycerol for slide preparation and imaged using a confocal microscope.

#### Detection of Extracellular Vap33:

To detect extracellular Vap33, we adapted our immunostaining protocol based on a previous method. Wild-type embryos were treated without detergents, substituting PBS for TBS, while maintaining the same anti-Vap33 concentration and incubation duration.

#### Primary Neurons:

Primary neurons were stained using a protocol similar to that for dissected embryos, but neurons were incubated with the secondary antibody for 1 hour at room temperature. We used anti-myc antibody (Developmental Studies Hybridoma Bank; 1:100). After staining, the neurons were submerged in PBS for imaging with a confocal microscope.

### Fluorescence Imaging

Fluorescence images were acquired using an inverted microscope (Ti-E, Nikon) equipped with a 100×, 1.45 NA oil immersion objective (Plan Apo, Nikon). The microscope was connected to a Dragonfly spinning disk confocal unit (CR-DFLY-501, Oxford Instruments). Three excitation lasers (488 nm, 561 nm, and 642 nm) were coupled to a multimode fiber passing through an Andor Borealis unit. A dichroic mirror (Dragonfly laser dichroic for 405/488/561/640 nm) and three bandpass filters (525/50 nm, 600/50 nm, and 725/40 nm) were placed in the imaging path. Images were recorded using an electron-multiplying charge-coupled device camera (Andor iXon, Oxford Instruments) controlled with Fusion software (Oxford Instruments).

For aCC imaging, optical sections with a thickness of 0.5 μm were acquired, spanning a z-depth of 8 μm from the surface of the ventral nerve cord (VNC), where aCC motoneurons are typically located. For VNC imaging, we focused on the dorsal region of the neuropile, where most motoneurons project their dendrites, covering a z-depth of 10 μm from the surface of the VNC. These images were reconstructed using ImageJ software from the National Institutes of Health (NIH). To maintain consistency, uniform laser power, gain, and exposure time settings were used during data collection. The figures display the resulting images as average intensity projections.

### Cell Culture and Transfection

*Drosophila* S2R + cells were maintained in SFX-INSECT culture medium (Hyclone) at 25°C. Cells were transfected with 300 ng of pACUH constructs using 2.5 μL of Effectene (Qiagen). To induce expression in transiently transfected cells, 300 ng of pAC-GAL4 (Addgene) was co-transfected. Transfections were carried out in 24-well plates. The cells were analyzed 48 hours post-transfection.

### Co-Immunoprecipitation Assay

S2 cells expressing *pACUH* constructs were lysed in 450 μL of 1% NP-40 lysis buffer. Lysates were incubated with 5 μL of Pierce Protein A/G Magnetic Beads (Thermo Fisher Scientific) overnight at 4°C with continuous rotation. The beads were washed three times with PBS and eluted with SDS loading buffer. Samples were subjected to SDS-PAGE using a 4–20% polyacrylamide gel (Bio-Rad), followed by protein transfer onto PVDF membranes (Thermo Fisher Scientific). The membranes were blocked at room temperature for 1 hour in 4% skimmed milk in TBS-T (0.1% Tween-20 in TBS) and probed with mouse anti-FLAG antibody (MilliporeSigma; 1:1000 dilution). Anti-mouse secondary antibodies conjugated to horseradish peroxidase (Thermo Fisher Scientific) were used at a dilution of 1:10,000 for 1 hour at room temperature. Blots were developed with a chemiluminescent substrate (Syngene) and imaged using the ChemiDoc Imaging System (Bio-Rad).

### Single-Cell RNA Sequencing Data Analysis

Single-cell RNA sequencing data were obtained from the NCBI Gene Expression Omnibus (GEO) under accession number GSE202987. Analysis was conducted using R scripts sourced from GitHub (https://github.com/AustinSeroka/2022-Doe-Drosophila-Embryo-Atlas). We employed the Seurat package (v3.1.2) in R, following standard protocols for quality control, data normalization, and analytical procedures. Cells were selected based on mitochondrial gene expression, retaining those with less than 20% mitochondrial reads for further analysis. The *sctransform* tool was used for data normalization to mitigate technical variance. Subsequent filtering and sub-clustering of the central nervous system (CNS) dataset were based on the expression of established neuronal markers, such as *elav*. Clusters were identified using a non-biased approach according to gene expression profiles. Gene expression levels are reported as normalized counts and categorized by developmental stage.

### Motoneuron Labeling and Imaging

To assess dendritic filopodia in aCC motoneurons, we performed targeted labeling using a lipophilic dye, focusing on four neurons within the A2–5 segments per embryo ^69^. Embryos were collected at 15:00 AEL, a time point selected because dendritic filopodia have stabilized and maintain a consistent number, facilitating reliable genotype comparisons. Although labeling efficiency varied due to the challenging injection process, typically 2–3 neurons per embryo were successfully labeled and included in the quantification.

### Quantification of Dendritic Filopodia

We quantified ipsilateral dendritic filopodia extending more than 1 μm along the axon shafts of labeled aCC motoneurons at 15:00 AEL. This time point was chosen because, unlike the dynamic branching observed at 13:00 AEL, the number of filopodia stabilizes, allowing for consistent comparisons across genotypes. Dendritic filopodia were counted using high-resolution confocal microscopy images, and the data were used to compare different genotypes.

### Confocal Microscopy and Imaging of Vap33

Extracellular Vap33 levels were quantified using confocal microscopy. Imaging focused on the dendritic regions of motoneurons, guided by the position of longitudinal connectives, as most motoneurons project dendrites dorsally within a 10 μm range of these connectives. For analysis, we concentrated on a single abdominal segment within the VNC and measured the average overall fluorescence intensity of Vap33. Measurements were obtained from wild-type embryos at 11:00, 13:00, and 15:00 AEL, as well as from *Vap33* mutants at 15:00 AEL. All fluorescence intensity values were normalized relative to wild-type and mutant embryos at 15:00 AEL, with values set to 0 and 1, respectively.

### FRET Imaging and Analysis

Fluorescence resonance energy transfer (FRET) data were acquired to measure Cdc42 activation in the proximal axon region of aCC motoneurons at 15:00 AEL. Imaging was performed using an upright Axio Imager Z2 microscope (Carl Zeiss) equipped with a 63× 1.4 NA oil immersion objective (Plan Apo, Carl Zeiss) and an LSM 880 scanning head with a 32-channel GaAsP spectral photomultiplier tube detector. The 458 nm line of an argon laser was used for excitation with the main beam splitter MBS-458/514. Emitted light was detected using two emission filters: 454–518 nm for cyan fluorescent protein (CFP) and 517–605 nm for yellow fluorescent protein (YFP). Image capture was controlled using Zen software (Carl Zeiss).

Raw images were processed by subtracting background fluorescence intensity from non-labeled cells to correct for background noise. Yellow/cyan emission ratios were calculated to generate “FRET images,” representing Cdc42 activation levels.

### Quantification of Cdc42 Activation

To quantify Cdc42 activation along aCC axons, FRET images underwent threshold segmentation to distinguish “object” pixels (representing aCC motoneurons) from “background,” based on predefined thresholds. This segmentation was applied across multiple z-planes captured for each neuron. The total yellow/cyan (Y/C) ratio values across all planes were divided by the volume to generate two-dimensional (2D) density maps (**Extended Data** Fig. 2b, top). From these maps, bar graphs were generated.

For detailed analysis, 30 μm-long lines perpendicular to the midline were drawn on each 2D density map. Values along these lines were averaged and plotted as line charts to represent the Y/C ratio over distance from the cell body (**Extended Data** Fig. 2b, bottom). To account for variations in protein expression among neurons, values were normalized based on the average Y/C ratio at the cell body (peak) and the axon shaft (baseline, located 20–25 μm from the cell body). Each line plot was fitted with a Gaussian equation, and the area under the curve was calculated to estimate the relative level of Cdc42 activation. This analysis was performed on 10 neurons per genotype, and the resulting values were averaged. Comparisons across genotypes or time points were presented as fold changes.

### Statistical Analysis

Statistical analyses were performed using OriginPro (OriginLab). Depending on data distribution, we applied Student’s *t*-test, Welch’s *t*-test, or the Mann–Whitney *U* test for pairwise comparisons. For multiple comparisons, one-way ANOVA followed by Tukey’s post hoc test was used. Data are presented as means ± standard errors of the means (SEMs) in all figures. Sample sizes for FRET analyses and filopodia counting were determined based on preliminary experiments and existing literature to ensure reproducibility. A power analysis, using standard deviations from preliminary data, indicated that a sample size of *n* = 11 neurons for dendrite counts and *n* = 10 neurons for distribution analysis would detect differences with 90% power at a 95% confidence level. Specific statistical tests used and sample sizes for each experiment are provided in the figure legends.

## Data Availability

All plasmids and transgenic flies created for this study are available upon request to the lead contact.
